# An Interactive Visualization for Feature Localization in Deep Neural Networks

**DOI:** 10.3389/frai.2020.00049

**Published:** 2020-07-23

**Authors:** Martin Zurowietz, Tim W. Nattkemper

**Affiliations:** Biodata Mining Group, Faculty of Technology, Bielefeld University, Bielefeld, Germany

**Keywords:** explainable deep learning, deep neural network visualization, visual analytics, interactive visualization, web application, computer vision, machine learning

## Abstract

Deep artificial neural networks have become the go-to method for many machine learning tasks. In the field of computer vision, deep convolutional neural networks achieve state-of-the-art performance for tasks such as classification, object detection, or instance segmentation. As deep neural networks become more and more complex, their inner workings become more and more opaque, rendering them a “black box” whose decision making process is no longer comprehensible. In recent years, various methods have been presented that attempt to peek inside the black box and to visualize the inner workings of deep neural networks, with a focus on deep convolutional neural networks for computer vision. These methods can serve as a toolbox to facilitate the design and inspection of neural networks for computer vision and the interpretation of the decision making process of the network. Here, we present the new tool Interactive Feature Localization in Deep neural networks (IFeaLiD) which provides a novel visualization approach to convolutional neural network layers. The tool interprets neural network layers as multivariate feature maps and visualizes the similarity between the feature vectors of individual pixels of an input image in a heat map display. The similarity display can reveal how the input image is perceived by different layers of the network and how the perception of one particular image region compares to the perception of the remaining image. IFeaLiD runs interactively in a web browser and can process even high resolution feature maps in real time by using GPU acceleration with WebGL 2. We present examples from four computer vision datasets with feature maps from different layers of a pre-trained ResNet101. IFeaLiD is open source and available online at https://ifealid.cebitec.uni-bielefeld.de.

## 1. Introduction

With the rapid increase in computing power over the past decade, deep artificial neural networks have become the go-to method for many machine learning tasks and achieve state-of-the-art performance in areas such as speech recognition, drug discovery, genomics, or computer vision (LeCun et al., 2015). The field of computer vision, in particular, quickly developed a wide range of methods based on neural networks for tasks such as image classification, object detection, or instance segmentation. One popular neural network architecture for computer vision is the convolutional neural network (CNN), which mimics the human visual pathway and can achieve impressive performance (Krizhevsky et al., 2012). One property that is inherent to all deep neural network architectures, including CNNs, is their high complexity owing to their very large number of internal parameters. For this reason, a CNN is generally regarded as “black box” whose inner working and decision making process is opaque (Wang et al., 2015; Yosinski et al., 2015; Rauber et al., 2016; Zintgraf et al., 2016; Samek et al., 2017; Chang et al., 2020). As CNNs became more and more popular, numerous techniques have been presented to facilitate the design and to understand the inner workings of a network through visualization (Seifert et al., 2017). Visualization techniques of CNNs can generally be filed into two categories: feature visualization and attribution (Olah et al., 2017).

**Figure 1:**
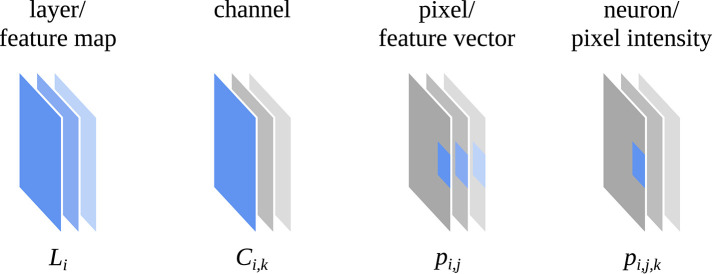
Definition of a layer (*L*_*i*_), channel (*C*_*i,k*_), pixel (*p*_*i,j*_), and neuron (*p*_*i,j,k*_) of a convolutional neural network that is used in this work. Adapted from Olah et al. (2017) (CC BY 4.0, https://distill.pub/2017/feature-visualization).

Feature visualization attempts to depict how a CNN encodes different image properties or, in other words, what (part of) a CNN “is looking for.” One of the methods for feature visualization is activation maximization (Erhan et al., 2009; Nguyen et al., 2016) which can be applied at different levels of a CNN, e.g., to a whole layer of the network, a single channel of a layer or a single neuron of a channel (see Figure 1). Among the most important discoveries through feature visualization with activation maximization is the fact that a CNN tends to build up its understanding of an image in a hierarchical way over many layers (Zeiler and Fergus, 2014; Olah et al., 2017). Lower layers respond to basic visual properties such as edges or textures, whereas higher layers respond to more abstract properties such as patterns, parts, or objects.

Attribution based methods make use of the fact that CNNs retain the spatial layout of the pixels of the input image throughout the different layers of the network. This particular trait is used in visualizations to highlight parts of an input image that are (most) responsible for the response of the network. Zeiler and Fergus (2014) used an occlusion approach to identify the regions of an image that contribute the most to a given network response. Zintgraf et al. (2016) extended this approach to visualize both image regions that act in favor and regions that act against a particular decision of the network. Stylianou et al. (2019) visualized, which regions of an input image are most salient for the decision that two images are similar. Most of the methods for attribution visualize salient image regions with a pseudo-color heat map which is transparently overlayed over the input image (Seifert et al., 2017). Such a heat map display can explain in an intuitive way why a network reaches a certain decision, e.g., which characteristics of the letter “3” identify it as such (Samek et al., 2016).

Various visualization techniques have been incorporated into interactive tools that aim to help in the design process of a CNN and facilitate the general interpretability of the network. Yosinski et al. (2015) presented two tools that visualize the activations of the layers of a CNN as well as feature visualizations of a selected channel in real time as the network processes an image or live video. Pezzotti et al. (2017) developed DeepEyes, a tool that combines many linked visualizations to monitor a network during training and to show how it changes over time. Kahng et al. (2017) presented ActiVis, an interactive visualization system that allows the interpretation and inspection of large-scale deep learning models and results, down to the level of individual neuron activations. Olah et al. (2018) developed interactive visualizations that combine feature visualization and attribution in an attempt to reduce the representations learned by an CNN to a human-comprehensible level. Spinner et al. (2019) presented the framework and interactive tool explAIner to facilitate the understanding, diagnosis, and refinement of machine learning models.

In this work, we present the new tool Interactive Feature Localization in Deep neural networks (IFeaLiD) which provides a novel visualization of CNN layers that shares characteristics of both feature visualization and attribution. The tool interprets CNN layers as multivariate feature maps and visualizes the similarity between the feature vectors of individual pixels of an input image in a heat map display. When used to compare different layers of a CNN, the visualization can highlight the hierarchically organized visual perception of a CNN with respect to a particular input image. Used only on a single layer, the visualization can point out which regions of an input image are perceived as similar by the network. These applications can be filed into the two research directions “understanding” and “debugging” of explainable deep learning as defined by Choo and Liu (2018). In contrast to many related approaches to visualize deep neural networks, the visualization of IFeaLiD is not limited to networks for the classification of images but can be applied to any CNN for computer vision (e.g., for tasks such as object detection or segmentation). IFeaLiD is implemented as a web application and the interactive visualization runs in real time in a web browser.

To illustrate possible applications, we present use cases for three different scenarios in which IFeaLiD could be applied:

**Use Case 1:** A computer vision novice seeks an intuitive understanding of how a CNN perceives images and how the perception changes through subsequent network layers. They work either on their own or as part of a lecture/course on machine learning for computer vision.
**Use Case 2:** A computer vision expert collaborates with other researchers such as biologists or medical experts for interdisciplinary research. The computer vision expert wishes to convey a basic understanding of the visual perception of CNNs to facilitate a productive discussion about the applications in their field of research.
**Use Case 3:** A computer vision researcher develops a new CNN architecture and wishes to investigate certain input images that cause an unintended network response. They want to inspect the output of individual layers of the network for the input images in order to understand the unintended behavior.

The remaining paper is structured as follows: In section 2, we describe the detailed process to obtain the visualization of IFeaLiD, using feature maps generated by ResNet101 (He et al., 2016) as example. We present relevant implementation details of the web application and show the final application interface. In section 3, we present example visualizations from the four computer vision datasets Cityscapes (Cordts et al., 2016), COCO (Lin et al., 2014), DIV2K (Agustsson and Timofte, 2017), and DOTA (Xia et al., 2018), obtained with ResNet101. We conclude the paper in section 4 with a discussion about the relevance and possible applications of IFeaLiD and the novel visualization with a special focus on the three presented use cases. IFeaLiD is open source and available online^1^.

## 2. Method

The IFeaLiD tool provides a visualization of a CNN layer which runs interactively in a web browser. For the visualization, a CNN layer is interpreted as multivariate feature map and pixels are colored according to the similarity of their feature vectors to the feature vector of a selected reference pixel. As a web application written in PHP and JavaScript, IFeaLiD can be used on many platforms and visualizations can be easily shared. In the following section, we define the interpretation of a CNN layer as feature map and describe how the data is transformed to allow processing by JavaScript in a web browser. Next, we show how the similarity between pixel feature vectors is computed and how real time processing of even high resolution feature maps is achieved by leveraging GPU acceleration with WebGL 2. Finally, we present the user interface of the web application.

### 2.1. Feature Map Extraction

A typical CNN for computer vision such as ResNet101 (He et al., 2016) processes an input image *L*_0_ with a width of *w*_0_, height of *h*_0_ and number of channels *d*_0_ (usually with *d*_0_ = 3 color channels) through a chain of *n* layers with the layer outputs {*L*_*i*_ | 1 ≤ *i* ≤ *n*}. Each layer output consists of pixels *L*_*i*_ = {*p*_*i,j*_ | 1 ≤ *j* ≤ *w*_*i*_ × *h*_*i*_} and each pixel consists of intensity values *p*_*i,j*_ = {*p*_*i,j,k*_ | *p*_*i,j,k*_ ∈ ℝ, 1 ≤ *k* ≤ *d*_*i*_}. Often, a layer output is also described as a set of channels *L*_*i*_ = {*C*_*i,k*_ | 1 ≤ *k* ≤ *d*_*i*_} where each channel consists of the pixel intensity values *C*_*i,k*_ = {*p*_*i,j,k*_ | 1 ≤ *j* ≤ *w*_*i*_ × *h*_*i*_} (see Figure 1).

**Figure 2:**
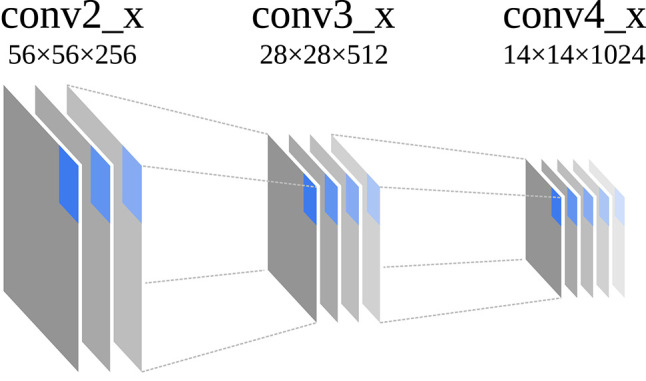
Schematic building blocks and their original dimensions (*w*_*i*_ × *h*_*i*_ × *d*_*i*_) of the different stages of ResNet101 that were used to extract feature maps in this work. Original dimensions are as reported by He et al. (2016). *w*_*i*_ and *h*_*i*_ vary depending on the dimension of the input image. One pixel of each layer is highlighted that corresponds to the same location in the input image.

In most cases, the spatial input image resolution *w*_0_ × *h*_0_ is successively downsampled by convolution operations with a stride greater than two or pooling operations, resulting in *w*_*i*_ > *w*_*q*_ and *h*_*i*_ > *h*_*q*_ for *i* < *q*. A pixel of the input image *L*_0_ can always be mapped to a pixel of a layer output *L*_*i*_ and vice versa, as the spatial layout of the pixels is preserved by downsampling and pooling. At the same time as the spatial resolution is reduced, the channel resolution is increased so that *d*_*i*_ < *d*_*q*_ for *i* < *q* (cf. Figure 2). As with many other CNN architectures, ResNet101 was originally applied for the task of image classification. For this reason, the final layer *n* was connected to a fully convolutional layer to produce a vector of class probabilities for a given input image. However, such a CNN can also be used as a feature extractor, interpreting the layer output *L*_*i*_ as multivariate feature map and the pixels *p*_*i,j*_ as feature vectors for a given input image *L*_0_.

IFeaLiD uses the interpretation of *L*_*i*_ as a feature map to visualize the output of a CNN layer. The visualization is rendered in real time with JavaScript and WebGL 2 in a web browser. The transfer of data from the layer output of a CNN to a JavaScript application and WebGL 2 in a web browser is not straight forward. Popular machine learning libraries such as TensorFlow (Abadi et al., 2016) for Python typically return output in the form of a NumPy array (Walt et al., 2011) of 32 bit floating point values. In the case of a feature map *L*_*i*_, the NumPy array has a shape of (*w*_*i*_, *h*_*i*_, *d*_*i*_). WebGL 2 is designed to enable the GPU accelerated display of three-dimensional graphics such as games with JavaScript in the browser. To this end, two-dimensional data such as images can be stored and processed in the form of “textures,” which are usually two-dimensional arrays of four 8 bit unsigned integers (i.e., three color channels and one alpha channel). While WebGL 2 is also capable of storing 32 bit floating point textures, the limitation to a maximum of four channels per texture remains.

**Figure 3:**
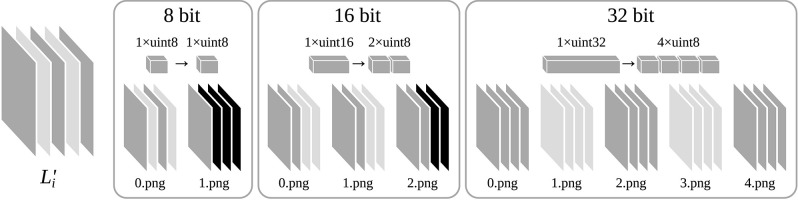
Example of a transformed feature map ${L^{\prime }}_{i}$ with *d*_*i*_ = 5 channels that is split into multiple PNG files for datasets of 8, 16, and 32 bit numeric precision, respectively. Each PNG file consists of four channels of 8 bit unsigned integers. 16 bit unsigned integers of the feature map are packed into two and 32 bit unsigned integers are packed into four 8 bit unsigned integers to be stored in one PNG file.

In order to efficiently transfer a feature map *L*_*i*_ with an arbitrary number of channels *d*_*i*_ from a NumPy array to a WebGL 2 texture, we have developed a method that splits up a NumPy array of 32 bit floating point values into a set of PNG images. Just like a WebGL texture, a PNG image is able to losslessly store a two-dimensional array of four 8 bit unsigned integers and is natively supported by web browsers and JavaScript. One PNG image can store the intensity values of one channel *C*_*i,k*_, by packing each 32 bit value into four 8 bit unsigned integers. This way, a feature map *L*_*i*_ can be stored in a dataset of *d*_*i*_ PNG images (see Figure 3). For reasons of reduced dataset size and higher processing speed, IFeaLiD also supports datasets with a reduced numeric precision of 16 bit or 8 bit. A 16 bit value is packed into two 8 bit unsigned integers and 8 bit values are used unchanged. A feature map *L*_*i*_ with 16 bit precision can be stored in ⌈0.5·*d*_*i*_⌉ PNG images and a feature map with 8 bit precision can be stored in ⌈0.25·*d*_*i*_⌉ PNG images (see Figure 3). In order to process both 32 bit, 16 bit, and 8 bit precision datasets in the same way, the 32 bit and 16 bit floating point values *p*_*i,j,k*_ are transformed to 32 bit and 16 bit unsigned integers $p_{i,j,k}^{\prime }$ (see Equation 1), producing the transformed feature map ${L^{\prime }}_{i}$.

(1) $${p^{\prime }}_{i,j,k}=\left\lceil \frac{p_{i,j,k}-\min_{j,k}\;p_{i,j,k}}{\max_{j,k}\;p_{i,j,k}-\min_{j,k}\;p_{i,j,k}}\cdot \;p_{\max}\right\rceil$$

(2) $$p_{\text{max}}\text{=}\{\begin{array}{ll} \text{ 255,} & \text{ 8 bit precision}\\ \text{ 65535,} & \text{ 16 bit precision}\\ \text{4294967295,} & \text{ 32 bit precision} \end{array}$$

### 2.2. Interactive Visualization With WebGL 2

The visualization of IFeaLiD displays the similarity between individual pixels of a feature map as a heat map. The similarity value *s*_*i,j*_ of a pixel at index *j* is computed based on the angular similarity, which is the inverse angle distance between the pixel's feature vector and the feature vector of a selected reference pixel at index *r* (see Equation 3). The computation is performed based on the pixel intensity values $p_{i,j,k}^{\prime \prime }$ which are floating point values that were reconstructed from the unsigned integer representation $p_{i,j,k}^{\prime }$ (see Equation 4). The angle distance was chosen as it provides more distinct distances between very high dimensional feature vectors in this particular application. Other distances such as the Manhattan distance (*L*_1_ norm) or Euclidean distance (*L*_2_ norm) suffer from the “curse of dimensionality” (Bellman, 1956), providing little difference in the distances between high dimensional feature vectors (see Figure S1). They have been found unsuitable as distance metrics for data spaces with more than ten dimensions (Weber et al., 1998; Beyer et al., 1999). Aggarwal et al. (2001) suggest to use a fractional *L*_*k*_ norm for high dimensional data but how to choose the fractional *k* is not straight forward.

(3) $$s_{i,j}=1-\frac{2}{\pi }\cdot \cos^{-1}\frac{{p^{\prime }}_{i,j}•\;{p^{\prime \prime }}_{i,r}}{\left|\;{p^{\prime }}_{i,j}\;|\cdot |\;{p^{\prime \prime }}_{i,r}\;\right|}$$

(4) $${p^{\prime \prime }}_{i,j,k}=\frac{{p^{\prime }}_{i,j,k}}{p_{\max}}$$

In order to compute the visualization for changing reference pixels *p*_*i,r*_ in real time, GPU accelerated processing is essential. At the time of writing, WebGL is the only way for a JavaScript web application to perform sophisticated GPU accelerated computations. WebGL 2 is the newest version of the WebGL API that is available in most modern web browsers and includes features such as floating point textures or 32 bit and 16 bit unsigned integer data types. As WebGL is intended to be used for rendering three-dimensional scenes such as games, its use is limited to strictly specified rendering pipelines. To compute the visualization of IFeaLiD using WebGL, it must be implemented as such a rendering pipeline. A basic WebGL rendering pipeline consists of four steps: data input, vertex shader computation, fragment shader computation, and data output.

**Figure 4:**
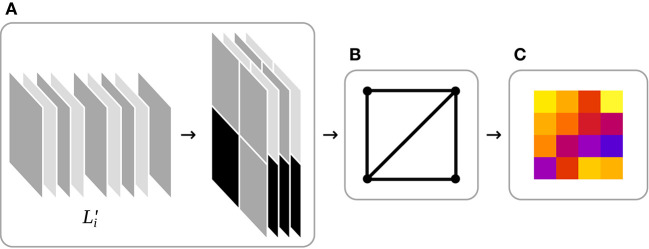
Schematic WebGL rendering pipeline of IFeaLiD. **(A)** Data input: Each four consecutive channels of the transformed feature map ${L^{\prime }}_{i}$ are stacked into tiles which are then arranged in a texture to approximate a square. **(B)** Vertex shader: The vertex shader positions a two-dimensional rectangle in the three-dimensional space of the WebGL scene. **(C)** Fragment shader: The fragment shader reconstructs the pixel intensity values ${p^{\prime \prime }}_{i,j,k}$ from their unsigned integer representation ${L^{\prime }}_{i}$ in the texture and computes the heat map visualization.

In the first step of a WebGL rendering pipeline, data such as vertex arrays, variables, or images are loaded into GPU memory. Vertex arrays represent the three-dimensional objects that should be rendered, variables can be used for any purpose and images are mostly two-dimensional four-channel arrays of 8 bit unsigned integers which are loaded into the GPU texture memory. In IFeaLiD, the dataset of a feature map is stored in texture memory. While WebGL 2 supports a wide range of texture data types, including the 8, 16, or 32 bit unsigned integers of a dataset, texture memory is always limited to four channels. To accommodate a dataset with an arbitrary number of channels, each four consecutive channels of the transformed feature map ${L^{\prime }}_{i}$ are stacked to a “tile” and the tiles are stored in a grid in texture memory to approximate a square (see Figure 4A).

After data input, the vertex shader computation is executed. A vertex shader determines the position and orientation of objects of a scene in three-dimensional space. In the case of IFeaLiD, the visualization is only two-dimensional and the vertex shader renders only a two-dimensional rectangle on which the visualization is projected in the next step (see Figure 4B).

In the third step, a fragment shader is executed to determine the color of each pixel of the final image that should be rendered. At this step, the pixel intensity values $p_{i,j,k}^{\prime \prime }$ are reconstructed from the unsigned integer representation $p_{i,j,k}^{\prime }$ in texture memory and the similarity value *s*_*i,j*_ is computed for each pixel of the feature map (see Equation 3). Next, the raw similarity values *s*_*i,j*_ are transformed to $s_{i,j}^{\prime }$ using the adaptive color scale optimization of Elmqvist et al. (2010), which optimizes the contrast of the heat map display of a given reference pixel (see Equation 5). Finally, a color map is applied to the optimized similarity values to produce the heat map visualization that is returned in the fourth step of the WebGL rendering pipeline (see Figure 4C).

(5) $${s^{\prime }}_{i,j}=\frac{s_{i,j}-\min_{j}s_{i,j}}{\max_{j}s_{i,j}-\min_{j}s_{i,j}}$$

### 2.3. Application Interface

**Figure 5:**
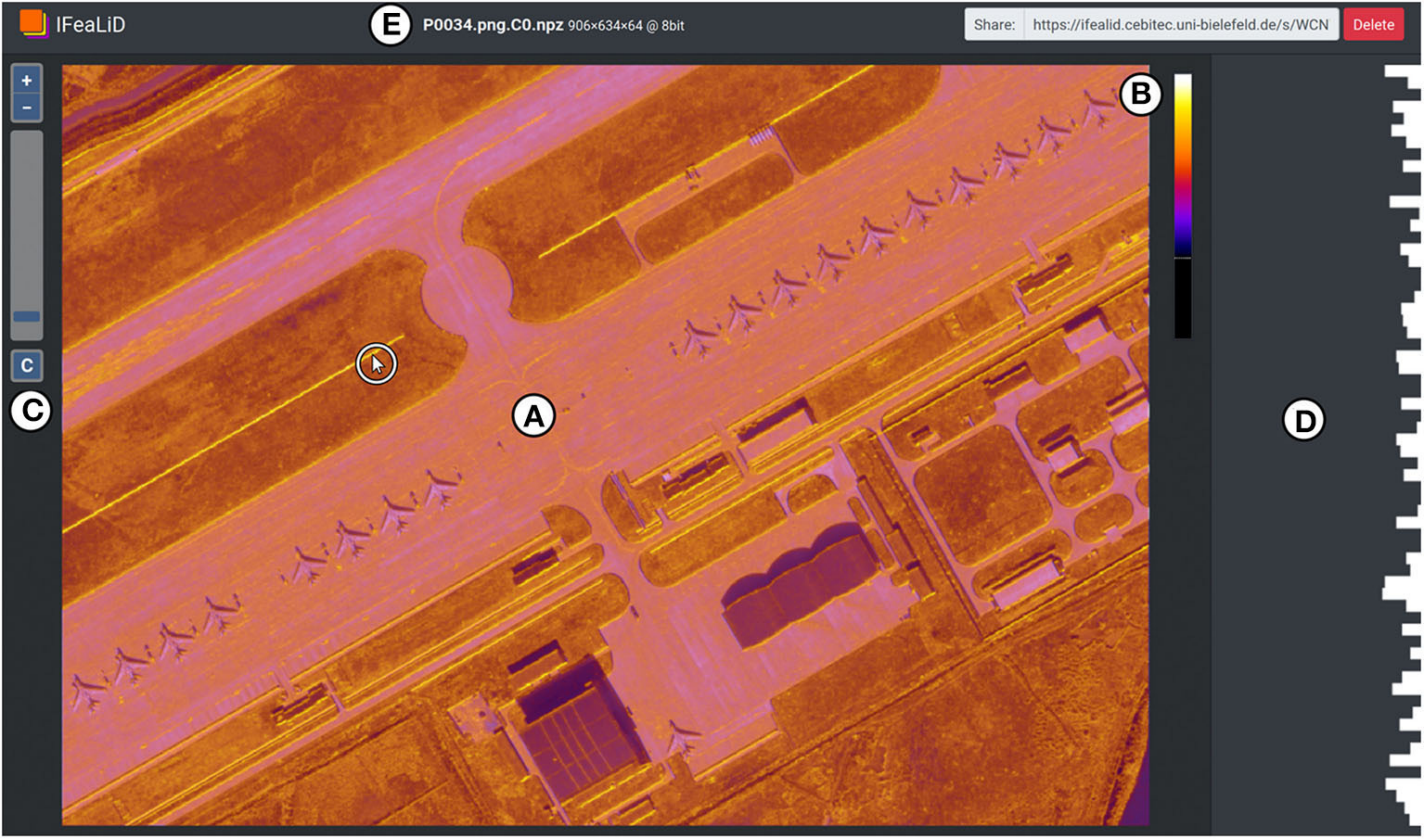
User interface of IFeaLiD with the feature map visualization of an image of the DOTA dataset (Xia et al., 2018). **(A)** The main display shows the heat map visualization based on the currently selected reference pixel *p*_*i,r*_ (marked with a cursor in a white circle). **(B)** Color scale which also visualizes the current effect of the color scale optimization as it does not fill the entire height of the element. **(C)** Slider control to adjust the opacity of the heat map visualization and button to switch between grayscale and color mode of the original image. **(D)** Sidebar with the bar chart visualization of the feature vector of the current reference pixel. **(E)** Top bar with dataset information and share URL.

In addition to the heat map visualization, the user interface of IFeaLiD provides further elements and interactions that enable the efficient and intuitive exploration of a feature map. The main display (see Figure 5A) shows the heat map visualization. The reference pixel *p*_*i,r*_ is selected interactively by moving the mouse over the heat map (see white cursor in Figure 5) and the visualization is updated in real time. A color scale is shown at the right of the main display (see Figure 5B) which also visualizes the current effect of the color scale optimization by stretching of the color scale. Optionally, the original input image *L*_0_ can be included in a dataset. If present, the input image is displayed beneath the heat map visualization and the opacity of each pixel of the heat map is initially set to the current similarity value $s_{i,j}^{\prime }$ of the pixel. A slider control is displayed at the left of the main display (see Figure 5C) which can be used to shift the opacity of each pixel in the range of $\left[s_{i,j}^{\prime },\;1\right]$. By default, the input image is displayed in grayscale so the colors do not interfere with the heat map visualization. With a click on a button (see Figure 5C), the input image can be switched between grayscale and color mode.

**Figure 6:**
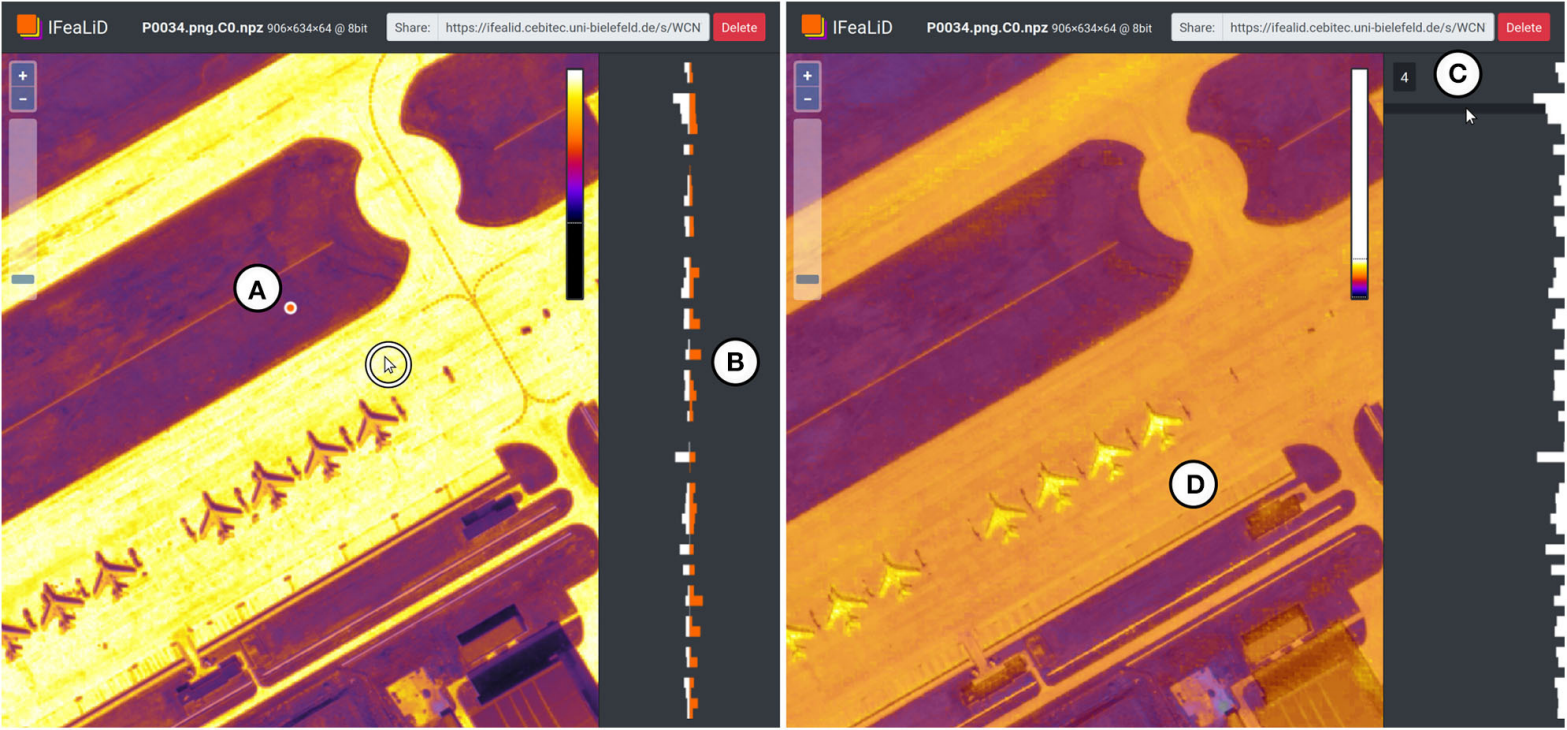
Additional interactions in IFeaLiD with the feature map visualization of an image of the DOTA dataset (Xia et al., 2018). **(A)** The position of a pinned reference pixel is marked with an orange dot. **(B)** In the bar chart visualization, the feature vector of the pinned reference pixel (orange) can be compared to the feature vector of the current reference pixel (white, position marked with a cursor in a white circle). **(C)** A channel of the feature map can be selected by hovering the mouse over the bar chart visualization of the feature vector. **(D)** The main display shows the pixel intensity values of the selected channel, to which color scale optimization and the color map was applied.

The sidebar (see Figure 5D) displays a bar chart visualization of the feature vector of the current reference pixel *p*_*i,r*_. To visually compare two feature vectors, a reference pixel can be pinned with a mouse click (see Figure 6A) and the feature vector of the pinned reference pixel is displayed continuously in the sidebar (see Figure 6B). If the mouse is subsequently moved over the heat map visualization, the bar chart visualizations of the pinned reference pixel and the current changing reference pixel can be compared. In addition, the mouse can be moved over the rows of the bar chart visualization (see Figure 6C) to interactively display the pixel intensity values of the *k*-th channel *C*_*i,k*_ of the feature map instead of the heat map visualization in the main display (see Figure 6D). This single channel visualization also applies the adaptive color scale optimization and color map to the pixel intensity values of the channel, which is described in the previous section.

The top bar of the user interface displays the dataset name, additional information such as the dimensions *w*_*i*_, *h*_*i*_, and *d*_*i*_ of the dataset, as well as an URL that can be used to share the visualization with others (see Figure 5E).

## 3. Results

To demonstrate applications for IFeaLiD, we present example visualizations of images from four different computer vision datasets. The visualizations are based on feature maps that were extracted at three different stages of ResNet101. In the following section we describe the setup that was used to obtain the visualizations and continue with a description of the examples.

### 3.1. Example Setup

The feature maps for the example visualizations were obtained by using ResNet101 (He et al., 2016) in the implementation of Abdulla (2017). The network was initialized with weights that were acquired through training on the COCO dataset (Lin et al., 2014), which are also provided by Abdulla (2017). The network was applied to each example image and the layer outputs of the last layer of each of the conv2_x, conv3_x and conv4_x stages were extracted as feature maps (cf. Figure 2). Accordingly, we refer to the feature maps as conv2_x, conv3_x and conv4_x in the following sections. Each feature map was extracted as NumPy array and converted to the IFeaLiD dataset format with 8 bit numeric precision as described in section 2.1. The datasets were uploaded to IFeaLiD and explored in the Firefox browser using a consumer laptop with an Intel® i7-7500U CPU (Intel® HD-Graphics 620).

### 3.2. Example Visualizations

**Figure 7:**
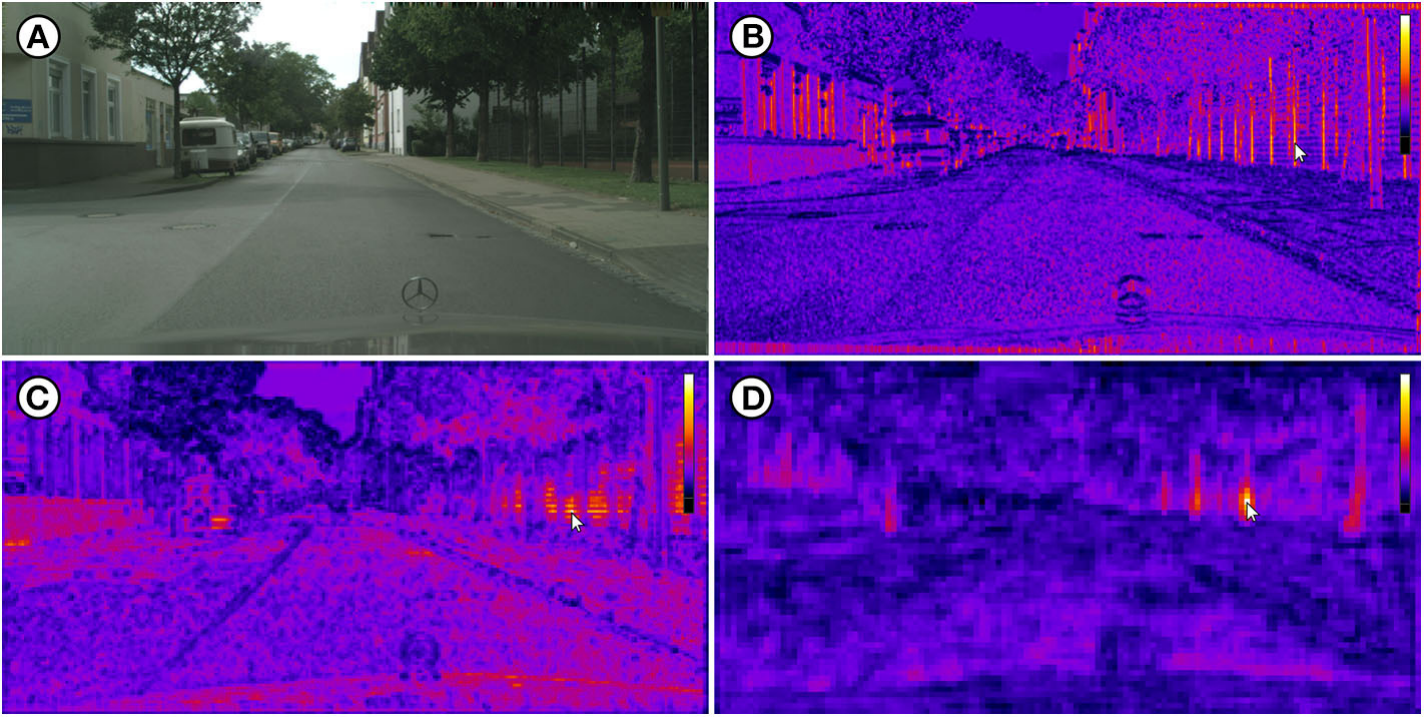
**(A)** Image bielefeld_000000_007186_leftImg8bit.png of the Cityscapes dataset (Cordts et al., 2016). **(B)** Visualization of the conv2_x feature map which shows edge activations. **(C)** Visualization of the conv3_x feature map which shows texture activations. **(D)** Visualization of the conv4_x feature map which shows object part activations (tree trunks). Each reference pixel *p*_*i,r*_ is marked with a cursor. Image reproduced with permission from Daimler AG, MPI Informatics, and TU Darmstadt (https://www.cityscapes-dataset.com).

**Figure 8:**
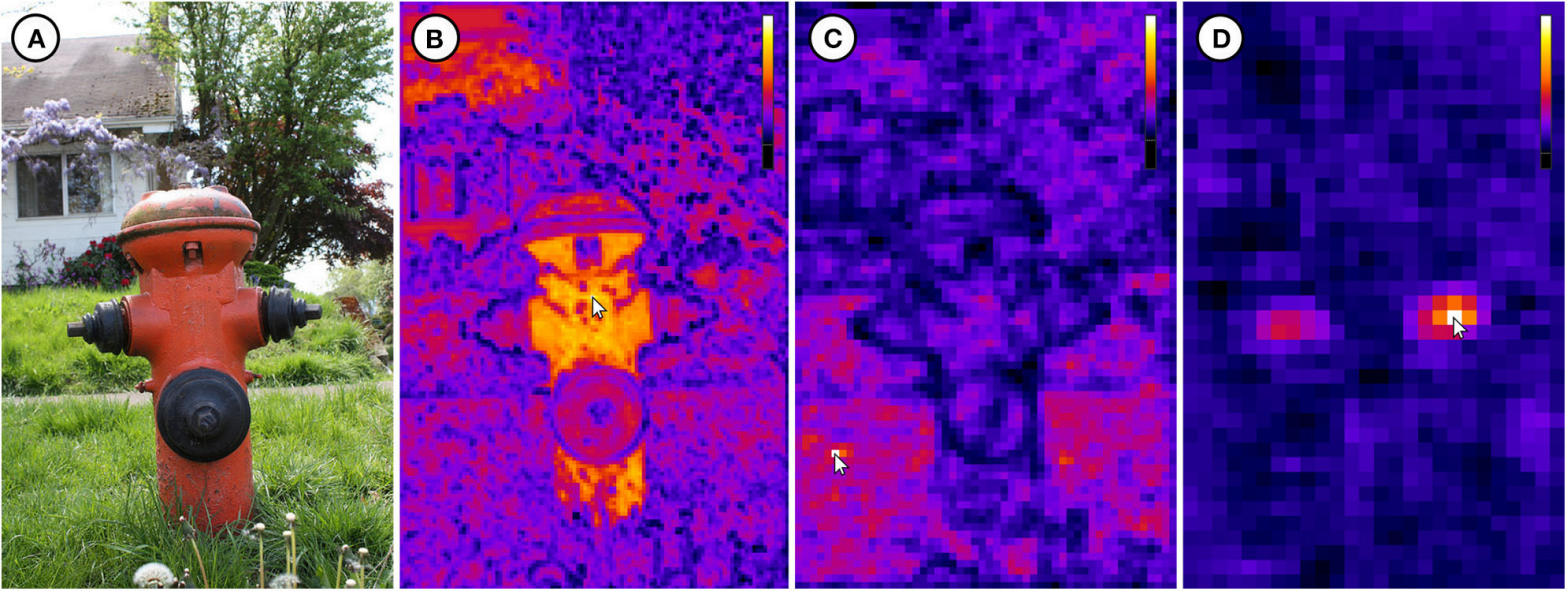
**(A)** Image 000000015746.jpg of the COCO dataset (Lin et al., 2014). **(B)** Visualization of the conv2_x feature map which shows color activations. **(C)** Visualization of the conv3_x feature map which shows texture activations. **(D)** Visualization of the conv4_x feature map which shows object part activations (side valve). Each reference pixel *p*_*i,r*_ is marked with a cursor. Image ©2009 by Flickr user piddix (CC BY 2.0, https://flic.kr/p/6mScoN).

**Figure 9:**
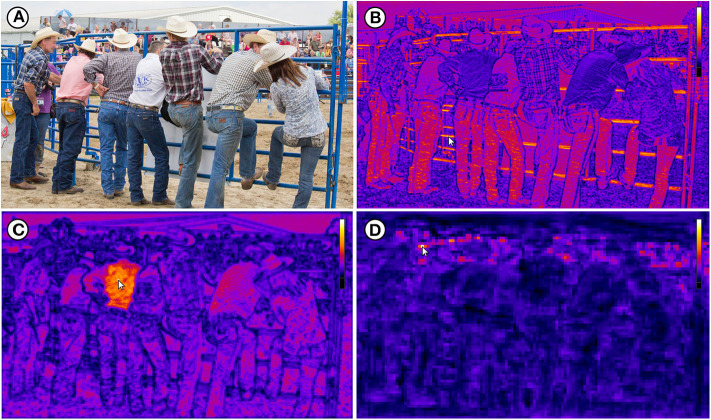
**(A)** Image 0804.png of the DIV2K dataset (Agustsson and Timofte, 2017). **(B)** Visualization of the conv2_x feature map which shows edge activations. **(C)** Visualization of the conv3_x feature map which shows texture activations. **(D)** Visualization of the conv4_x feature map which shows object activations (faces). Each reference pixel *p*_*i,r*_ is marked with a cursor. Image ©2014 by Flickr user cjuneau (CC BY 2.0, https://flic.kr/p/odGqwg), faces have been pixelated.

**Figure 10:**
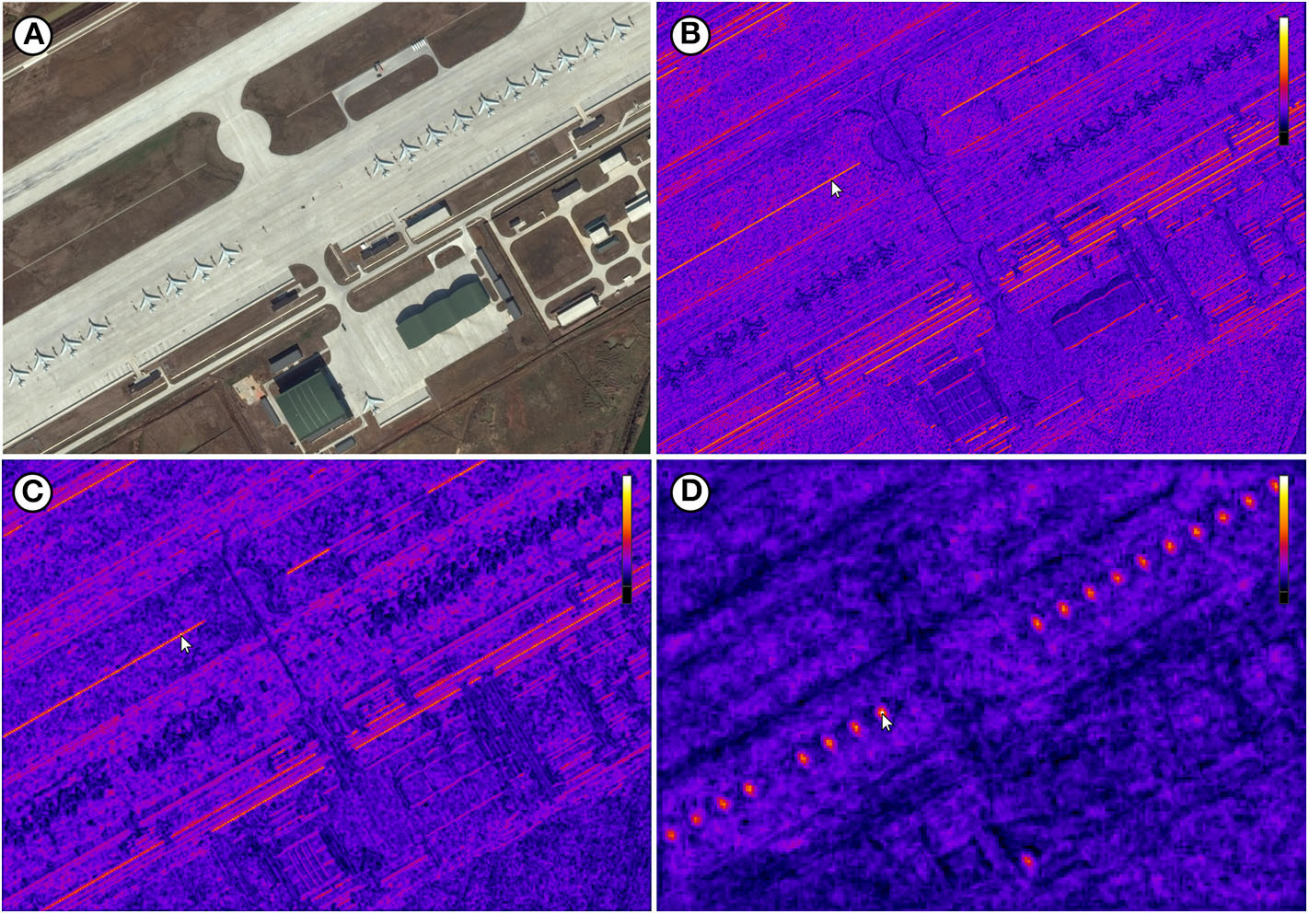
**(A)** Image P0034.png of the DOTA dataset (Xia et al., 2018). **(B)** Visualization of the conv2_x feature map which shows edge activations. **(C)** Visualization of the conv3_x feature map which still shows edge activations. **(D)** Visualization of the conv4_x feature map which shows object activations (planes). Each reference pixel *p*_*i,r*_ is marked with a cursor. Image ©2019 Google Earth.

The example visualizations are based on one image of each of the four computer vision datasets Cityscapes (Cordts et al., 2016), COCO (Lin et al., 2014), DIV2K (Agustsson and Timofte, 2017), and DOTA (Xia et al., 2018). Figures 7–10 show the original image *L*_0_ as well as IFeaLiD visualizations of the feature maps conv2_x, conv3_x and conv4_x for each example. For each visualization, a descriptive reference pixel *p*_*i,r*_ was selected to highlight specific properties of the feature maps. The visualizations are best viewed interactively. Dataset files and links to the interactive visualizations in IFeaLiD can be found in Zurowietz (2020). With the exception of the image of the COCO dataset, all example images have a high resolution, producing feature maps with 10^7^ to 10^8^ pixel intensity values (see Table 1). Even without a dedicated GPU on a consumer laptop, all visualizations were rendered and updated in real time without noticeable delay.

**Table 1:** Statistics of the images of the four computer vision datasets that were used as examples in this work, with the dimensions of the original image, the dimensions of each ResNet101 feature map and the total size of each feature map (*w*_*i*_·*h*_*i*_·*d*_*i*_).

**Dataset/Image**	**Feature map**	***w*_*i*_**	***h*_*i*_**	***d*_*i*_**	***w*_*i*_·*h*_*i*_·*d*_*i*_**
	*L*_0_	2, 048	1, 024	3	0.6·10^7^
Cityscapes (Cordts et al., 2016) /	conv2_x	512	256	256	3.4·10^7^
bielefeld_000000_007186_leftImg8bit.png	conv3_x	256	12	512	1.7·10^7^
	conv4_x	128	64	1, 024	0.8·10^7^
	*L*_0_	427	640	3	0.1·10^7^
COCO (Lin et al., 2014) /	conv2_x	106	160	256	0.4·10^7^
000000015746.jpg	conv3_x	52	80	512	0.2·10^7^
	conv4_x	26	40	1, 024	0.1·10^7^
	*L*_0_	2, 040	1, 200	3	0.7·10^7^
DIV2K (Agustsson and Timofte, 2017) /	conv2_x	510	300	256	3.9·10^7^
0804.png	conv3_x	254	150	512	2.0·10^7^
	conv4_x	126	74	1, 024	1.0·10^7^
	*L*_0_	3, 626	2, 542	3	2.8·10^7^
DOTA (Xia et al., 2018) /	conv2_x	906	634	256	14.7·10^7^
P0034.png	conv3_x	452	316	512	7.3·10^7^
	conv4_x	226	158	1, 024	3.7·10^7^

*The feature maps of the DOTA image are an order of magnitude larger than those of the remaining images*.

The IFeaLiD visualizations of the conv2_x stage of ResNet101 reveal similar feature vectors for similar gradients such as fence posts (see Figure 7B), bars (see Figure 9B), and lines (see Figure 10B) as well as similar colors (see Figure 8B). The feature maps of the conv3_x stage show similar feature vectors for similar textures such as a fence lattice (see Figure 7C), grass (see Figure 8C), or a checkered shirt (see Figure 9C). Notably, seemingly dissimilar parts of the image of the Cityscapes dataset show similar feature vectors (cf. fence lattice and the lower part of the trailer in Figure 7C). On the other hand, supposedly similar parts of the image of the DIV2K dataset show dissimilar feature vectors (cf. the different checkered shirts in Figure 9C). The visualizations of the conv4_x stage show similar feature vectors for similar parts such as tree trunks (see Figure 7D) or valves (see Figure 8D) as well as similar objects such as faces (see Figure 9D) or planes (see Figure 10D).

## 4. Discussion

IFeaLiD provides a novel visualization of deep neural network layer outputs which are interpreted as multivariate feature maps. To efficiently compute the visualization based on high dimensional data in a web application, we have developed a dataset format that allows the transfer of multivariate data to a browser-based JavaScript application and implemented the computation with GPU acceleration through WebGL 2. The dataset format supports different numeric precisions and the visualization is rendered in real time even for high-resolution images. In addition, the visualization of IFeaLiD is not limited to networks for the classification of images but can be applied to any CNN for computer vision (e.g., for tasks such as object detection or segmentation).

The presented examples illustrate possible applications of IFeaLiD and demonstrate what kind of deep neural network characteristics such a visualization can show. All example visualizations highlight the hierarchically organized visual perception of the network (see Figures 7–10). The lower layers (conv2_x) show similar activations for basic visual properties such as edges or gradients. The next higher layers (conv3_x) show similar activations for textures or patterns. The highest layers of the presented examples (conv4_x) show similar activations for whole objects or parts of objects. This is consistent with the hierarchy presented and visualized by Olah et al. (2017). As the visualization is less abstract than other approaches (e.g., feature visualization), it can be more intuitive for non-experts. This makes it well suited for the application in teaching where the visualization can be directly transferred back to the basic building blocks of a CNN (**Use Case 1**). In addition, it can easily be explained to non-experts in the context of interdisciplinary research (**Use Case 2**). The availability as a web application is another advantage as it requires no complex installation and allows easy sharing of visualizations with students or research collaborators.

Besides the comparison of feature maps of different layers of a network, the exploration of only a single feature map in IFeaLiD can reveal interesting insights as well. In the conv3_x feature map of the Cityscapes example, similar feature vectors are shown for the fence lattice and the lower part of the trailer (see Figure 7C). The similar activations are probably caused by the similar patterns of dark and bright lines that are present at both locations. This could be a hint of why a network would incorrectly classify the trailer as fence or the other way around. On the other hand, the visualization can reveal that image regions which are perceived as similar by a human observer can be perceived as highly different by a neural network. In case of the DIV2K example, the checkered shirt of the third person from the left that is selected with the reference pixel in Figure 9C shows highly different feature vectors than the similarly checkered shirt of the second person from the right. Even the arm of the person with the selected shirt shows different feature vectors. In the same vein, the side and front valves of the hydrant in the COCO example are not perceived as similar by the network in the conv4_x feature map, contrary to human intuition (see Figure 8). Insights such as these can facilitate the development of new CNN architectures as they help developers to understand unintended behavior of the CNN for certain instances of input images (**Use Case 3**).

Another valuable insight that the visualization provides for the presented examples is how pre-trained weights of a neural network can be reused and transferred to other datasets and visual domains. Although the weights that were used to initialize ResNet101 in our examples were produced through training on the COCO dataset only, the network layers produce plausible activations for the other three datasets as well. Even the feature maps of the DOTA example, which come from an entirely different visual domain than the everyday images of COCO, show plausible activations. This might not always be the case for all pre-trained weights and target datasets. IFeaLiD can be a way to quickly assess the reusability of pre-trained weights and to determine if they can be applied for a given target dataset. This can be valuable knowledge in cases where new applications of CNNs are explored (**Use Case 2** and **Use Case 3**).

While IFeaLiD is well-suited for the use cases described above, there are some limitations. For one, it cannot be easily used to get an overview over the learned representation of the network as a whole, since it only allows the inspection of one network layer at a time. Other visualization approaches, while being more abstract, can provide a more global overview. In our examples, the angular distance metric on which the visualization of IFeaLiD is based produced a better contrast for high dimensional data than other well known distance metrics such as the *L*_*k*_ norm (see Figure S1). However, the angular distance metric does not take the activation magnitudes of neurons into account, which could be important in certain cases. Ultimately, IFeaLiD could offer multiple distance metrics to choose from, which we leave as a topic for future work. The implementation of IFeaLiD as a web application allows easy and platform-independent access as well as sharing of visualizations with others. Compared with a classical desktop application, though, the web application may not be as performant, since a desktop application allows a more flexible and direct access to GPU acceleration. Still, in our tests, the implementation with WebGL 2 was always fast enough for a responsive and interactive visualization.

The presented examples show how the visualization of IFeaLiD can be a valuable tool to facilitate the understanding of the inner workings of a deep neural network. Used on a single feature map, the tool allows the localization of similar feature vectors for a given input image which could explain an unintuitive classification or detection output of the network. Used to compare multiple feature maps of the same network, the visualization can help to understand how the visual perception is organized in the network architecture. Finally, the visualization can be used to assess whether neural network weights that were obtained by training on one dataset can be reused for another dataset that potentially contains images of an entirely different visual domain. Readily available online as a web application, IFeaLiD is an easy to use and shareable addition to the toolbox for the understanding and inspection of deep neural networks for computer vision.

## Data Availability Statement

The datasets presented in this study can be found in online repositories. The example datasets presented in this work can be accessed and explored online (https://doi.org/10.5281/zenodo.3741485). The code of IFeaLiD is available at GitHub (https://github.com/BiodataMiningGroup/IFeaLiD).

## Author Contributions

MZ and TN contributed to all aspects of designing the software/method, preparing the paper, and reviewing it. MZ implemented the software/method. Both authors contributed to manuscript revision, read, and approved the submitted version.

## Conflict of Interest

The authors declare that the research was conducted in the absence of any commercial or financial relationships that could be construed as a potential conflict of interest.

## Footnotes

^1^https://ifealid.cebitec.uni-bielefeld.de
