# Supplementary material for: An Interactive Visualization for Feature Localization in Deep Neural Networks
Source: Front Artif Intell. 2020 Jul 23;3:49. doi: 10.3389/frai.2020.00049 (PMC7861262; doi:10.3389/frai.2020.00049)
Supplement: Supplementary file 1 [file Image_1.PDF]

frontiers

Supplementary Material

1 COMPARISON OF SIMILARITY METRICS

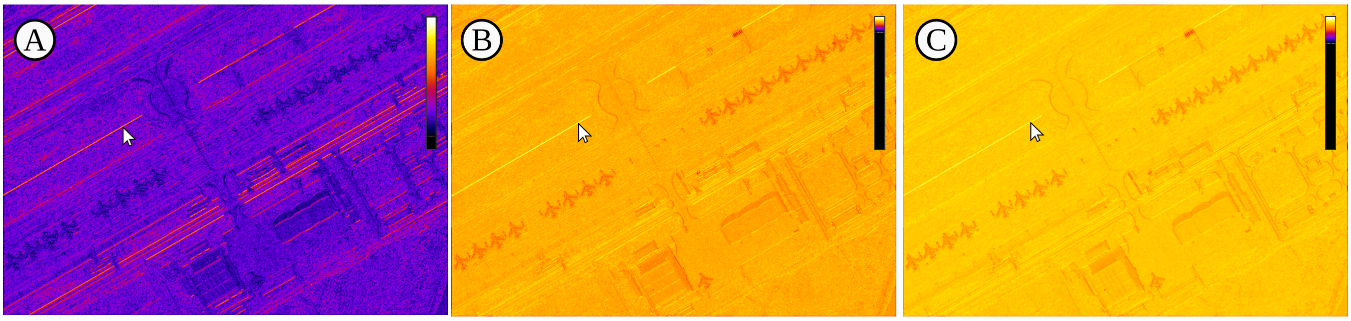

Figure S1. IFeaLiD visualizations of image `P0034.png` of the DOTA dataset (Xia et al., 2018) with different similarity measures. **(A)** angular similarity $s^{\mathrm{A}}_{i,j}$ (see Equation S1), **(B)** Manhattan similarity $s^{\mathrm{M}}_{i,j}$ (see Equation S2) and **(C)** Euclidean similarity $s^{\mathrm{E}}_{i,j}$ (see Equation S3). Each reference pixel $p_{i,r}$ is marked with a cursor. While all visualizations have a similar qualitative level (showing similar structures), the visualizations based on the Manhattan similarity and Euclidean similarity show a much lower contrast than the visualization based on the angular similarity. The visualization based on the angular similarity uses a much wider range of the available color map even though the color scale optimization is applied in all three cases (which is indicated by the color scales on the right of each image). A closer inspection of the visualizations reveals that the angular similarity shows a higher tolerance for outliers at the edges, possibly because it disregards the magnitude of neuron activations.

$$s^{\mathrm{A}}_{i,j} = 1 - \frac{2}{\pi} \cdot \cos^{-1} \frac{p''_{i,j} \bullet p''_{i,r}}{|\, p''_{i,j} \,| \cdot |\, p''_{i,r} \,|} \tag{S1}$$

$$s^{\mathrm{M}}_{i,j} = 1 - \frac{1}{d_i} \cdot \sum_k |p''_{i,j,k} - p''_{i,r,k}| \tag{S2}$$

$$s^{\mathrm{E}}_{i,j} = 1 - \sqrt{\frac{1}{d_i} \cdot \sum_k (p''_{i,j,k} - p''_{i,r,k})^2} \tag{S3}$$

REFERENCES

Xia, G.-S., Bai, X., Ding, J., Zhu, Z., Belongie, S., Luo, J., et al. (2018). Dota: A large-scale dataset for object detection in aerial images. In *Proceedings of the IEEE Conference on Computer Vision and Pattern Recognition*. 3974–3983
